# Development of a 3-transcript host expression assay to differentiate between viral and bacterial infections in pigs

**DOI:** 10.1371/journal.pone.0256106

**Published:** 2021-09-23

**Authors:** Bernt Hjertner, Claudia Lützelschwab, Elise Schieck, Benjamin Nzau, Sonal Henson, Marie Sjölund, Caroline Fossum, Ulf Magnusson

**Affiliations:** 1 Department of Biomedical Sciences and Veterinary Public Health, Swedish University of Agricultural Sciences, SLU, Uppsala, Sweden; 2 International Livestock Research Institute, Nairobi, Kenya; 3 Department of Animal Health and Antimicrobial Strategies, National Veterinary Institute, SVA, Uppsala, Sweden; 4 Department of Clinical Sciences, Swedish University of Agricultural Sciences, SLU, Uppsala, Sweden; George Washington University, UNITED STATES

## Abstract

Indiscriminate use of antibiotics to treat infections that are of viral origin contributes to unnecessary use which potentially may induce resistance in commensal bacteria. To counteract this a number of host gene transcriptional studies have been conducted to identify genes that are differently expressed during bacterial and viral infections in humans, and thus could be used as a tool to base decisions on the use of antibiotics. In this paper, we aimed to evaluate the potential of a selection of genes that have been considered biomarkers in humans, to differentially diagnose bacterial from viral infections in the pig. First porcine PBMC were induced with six toll-like receptor (TLR) agonists (FliC, LPS, ODN 2216, Pam3CSK4, poly I:C, R848) to mimic host gene expression induced by bacterial or viral pathogens, or exposed to heat-killed *Actinobacillus pleuropneumoniae* or a split influenza virus. Genes that were differentially expressed between bacterial and viral inducers were further evaluated on clinical material comprising eleven healthy pigs, and six pigs infected with *A*. *pleuropneumoniae*. This comprised three virally upregulated genes (*IFI44L*, *MxA*, *RSAD2*) and four bacterially upregulated genes (IL-1β, *IL-8*, *FAM89A*, *S100PBP*). All six infected pigs could be differentially diagnosed to healthy pigs using a host gene transcription assay based on the geometric average of the bacterially induced genes *IL-8* and *S100PBP* over that of the virally induced gene *MxA*.

## Introduction

Antimicrobial resistance is a globally emerging problem, threatening human and animal health [1]. Globally, the amount of antimicrobials used in the livestock sector exceeds that in the health sector and most of these antimicrobials are used in intensive pig rearing [2]. The latter is attributable to the practice of using antibiotics regularly for disease prevention and for promoting growth. Besides the requirement of veterinary prescription for sale, in 2006 the European Commission banned the use of all antimicrobials as feed additives for growth promotion [3] and some countries have selected restrictions on their use [4, 5]. However, especially in low- and middle-income countries such restrictive regulations are not in place or poorly enforced, and the use of antimicrobials is widespread and arbitrary partly because they are freely available without prescription [6, 7].

Also, in low- and high-income countries alike, the overuse of antibiotics in both human and veterinary medicine is partly due to inadequate treatments of viral diseases, misdiagnosed as bacterial infections [4]. Thus, a reliable and rapid diagnostic tool able to differentiate bacterial from viral infections would help to reduce the misuse of antibiotics in livestock.

Detection of acute-phase proteins in patient sera has traditionally been used to indicate ongoing bacterial infections in both human [8] and veterinary [9] medicine. However, the acute phase response is activated by several inflammatory conditions and not only by bacterial infections. The insight that the liver synthesizes acute-phase proteins in response to the cytokines IL-1β and IL-6 produced by macrophages during bacterial infections and that viral infections elicit production of type one interferons (IFN-α/β) introduced the detection of key cytokines in serum as indicators of on-going bacterial or viral infections in human medicine [10]. Today, the detection of biomarkers by bio- or immunoassays has been complemented by gene expression profiling, both in human and veterinary research [11, 12].

In the pig, we have previously evaluated acute-phase proteins and pro-inflammatory cytokines in studies of the efficacy of various antibiotics during experimental infections with the respiratory pathogen *Actinobacillus pleuropneumoniae* causing severe disease [13–15] and serum IFN-α as an indicator for ongoing viral infections [16–18]. These responses are clearly related to the type of infection but show a large individual variation and are relatively short-lived. Therefore, transcript signatures reflecting signaling pathways initiated via pattern recognition receptors (PRRs) are most likely better indicators, differentiating bacterial from viral infections.

Most studies on transcript signatures differentiating bacterial from viral infections has been done in humans. A 38-transcript signature differentially expressed in bacterially and virally infected febrile children, that was further reduced to a two-transcript signature based on the genes *FAM89A* (bacterial) and *IFI44L* (viral) has been described [19]. Based on biomarkers reviewed by Tsao et al. [12] and putative new biomarkers identified by Herberg et al. [19], a number of porcine counterparts were analyzed as potential differential biomarkers. These were first evaluated on porcine peripheral blood mononuclear cells (poPBMC) cultured in the presence of synthetic TLR agonists, heat-inactivated *A*. *pleuropneumoniae* and split influenza proteins, respectively. From this selection three virally upregulated genes (*IFI44L*, *MxA*, *RSAD2*) were further evaluated together with four genes supposedly indicative of bacterial infections (IL-1β, *IL-8*, *FAM89A*, *S100PBP*) on clinical samples. The aim of the present study was to, from the evaluated marker genes, identify a transcript signature enabling a differential diagnosis between bacterial and viral infections in the pig.

## Material and methods

### Experimental and clinical samples

For the first screenings of putative marker genes, heparinized blood was collected from clinically healthy specific pathogen free (SPF) pigs [20] housed at The Swedish Livestock Research Centre at the Swedish University of Agricultural Sciences, SLU, Uppsala, Sweden. Peripheral blood mononuclear cells (PBMC) were isolated by centrifugation on Ficoll Paque (Amersham Pharmacia Biotech, Uppsala, Sweden), and washed three times in PBS before resuspended in RPMI 1640 medium (BioWhittaker, Cambrex Bioscience, Verviers, Belgium), supplemented with HEPES (20 mM), L-glutamine (2 mM), penicillin (120 μg/mL), streptomycin (100 μg/mL), 2-mercaptoethanol (50 μM), and 5% fetal calf serum (Invitrogen, Life Technologies, Carlsbad, CA, USA). The PBMCs were seeded at 5–10 x10^6^ cells/well in 6 wells plates (Nunclon; Nunc, Roskilde, Denmark), and stimulated with the following TLR agonists: Pam3CSK4 (InvivoGen, San Diego, CA, USA), poly (I:C) (P-L Biochemicals, Milwaukee, Wis. USA), LPS from *E*. *coli* (SIGMA #L3024), FliC, VacciGrade^™^ (InvivoGen), R848 (InvivoGen), ODN 2216 (InvivoGen). Each agonists TLR specificity, “pathway” induced and final concentration is summarized in Table 1. Both ODN 2216 and poly (I:C) were transfected into the PBMC using Lipofectin^®^ (Invitrogen, 10μg/mL) according to the manufacturer instructions. After 18 h incubation at 37°C in a 7% CO_2_ atmosphere, cells were harvested for gene transcription analysis. As a control, PBMC cultured in plain growth medium were used. The PBMC were also exposed to 4.5x10^7^ heat-inactivated *A*. *pleuropneumoniae* serotype 2 collected during *in vitro* expansion [21, 22] or 5 μg/mL split influenza vaccine antigen (Vaxigrip 2014/2015: Sanofi Pasteur MSD, Diegem, Belgium).

**Table 1:** Summary of agonists.

Agonist	TLR specificity	“Viral inducer”	“Bacterial inducer”	Concentration (μg/mL)
Pam3CSK4	2/1		√	0,5
poly (I:C)	3	√		5
LPS	4		√	1
FliC	5		√	1
R848	7/8	√		5
ODN 2216	9	√		5

Porcine PBMC were exposed to different TLR agonists *in vitro* to evaluate the expression of a variety of bacterially and virally induced biomarkers. Listed is the toll-like receptor (TLR) specificity of each agonist, the dominant signalling pathway induced (viral or bacterial) and the final concentrations of agonists used.

For clinical studies, blood was collected in PAXgene^™^ Blood RNA Tubes (Becton, Dickinson and Company, NJ, USA) and stored at -80°C until RNA extraction. These blood samples were collected from six pigs originating from a herd declared free from major swine pathogens (SPF) (Serogrisen, Ransta, Sweden), experimentally infected with *A*. *pleuropneumoniae* serotype 2 (strain 700/89 National Veterinary Institute, Sweden). These pigs served as infected, non-treated control pigs in a study on benzylpenicillin dosage regimens [23] and samples were collected before necropsy at day 16 post-infection. At this time *A*. *pleuropneumoniae* was reisolated from four of the six pigs. All six pigs displayed macroscopically affected lung tissue. *A*. *pleuropneumoniae* was chosen as a model bacterial infection as it is one of the major bacterial pathogens causing respiratory disease in pigs, and because of vast previous experience from both *in vitro* and *in vivo* studies of the inflammatory response to it. As healthy controls, blood samples from eleven clinically healthy SPF pigs (Serogrisen, Ransta, Sweden) [24] were used.

The collection of blood for in vitro studies falls under the approval of Uppsala Ethical Committee on Animal Experiments (Dnr:C105214/15). The in vivo infection study was approved by the regional ethical committee in Uppsala (License C 37/16) and by the Swedish Medical Products Agency (License no. 5.1-2016-16737).

### Total RNA extraction and cDNA synthesis

Total RNA was extracted from all *in vitro* inductions by combining Trizol^®^ (Invitrogen, Carlsbad, CA, USA) with the column-based E.Z.N.A. total RNA kit (Omega Biotek, Norcross, GA, USA) according to Wikström et al. [25]. RNA from PAXgene tubes was isolated combining the PAXgene Blood RNA Kit with Trizol extraction. In brief, the PAXgene Blood RNA protocol was followed with the exception that Trizol was added instead of BR2 binding buffer and the sample went through one round of Trizol/chloroform extraction before adding ethanol and transfer to the spin column.

RNA quantity and purity was estimated by spectrophotometry (NanoDrop ND-1000, NanoDrop Technologies, Montchanin, DE, USA) and RNA quality index (RQI) was estimated using capillary gel electrophoresis (Experion RNA StdSense Analysis Kit, Bio-Rad Laboratories, Solna, Sweden). Total RNA (1.2 μg) from each sample were treated with RNAse-free DNAse I (Promega, Madison, WI, USA) followed by cDNA synthesis (GoScript Reverse transcription system; Promega, USA). Samples were diluted 5 × before storage at −20°C until qPCR analysis.

### Quantitative PCR

One or two assays targeting each gene selected from Herberg et al. [19] were designed and evaluated on porcine PBMC induced with different TLR agonists as well as the clinical samples from pigs experimentally infected with *A*. *pleuropneumoniae* serotype 2. The best performing pair of primers for each gene, whenever a positive result was obtained (data not shown), were optimized for primer concentration, annealing temperature, specificity and sensitivity. Three assays possibly indicative of viral infections (*IFI44L*, *IFIT3*, *RSAD2*) and three of bacterial infection (*FAM89A*, *SLPI*, *UPB1*) were selected. In addition, assays for *MxA* (viral) and *S100PBP* (bacterial) were designed and optimized. All other assays for interferon-related or pro-inflammatory genes were previously optimized [24]. Detailed primer information for all assays used is summarized in S1 Table.

Duplicate reactions of 2 μL cDNA in 23 μL of Quantitect SYBR Green PCR mix (Qiagen) were run in a CFX96 Touch PCR machine (Bio-Rad). The running conditions were as follow: an initial cycle of 95°C for 15 min followed by 40 cycles at 95°C for 15 s, the assay-specific annealing temperature for 30 s and 72°C for 30 seconds. Five reference genes (*PPIA*, *RPL32*, *HPRT*, *YWHAZ*, *GAPDH*) were assessed for their expression stability in all the samples using the geNorm software (qBasePLUS, Biogazelle). The stable expression of these genes was scored based on a gene stability parameter (*M*) and a coefficient of variation (C*V*), where *M* values < 0.5 and *CV* values < 0.2 indicate high expression stability. The most appropriate reference genes were accordingly selected for normalization of gene expression. The expression of each gene of interest was calculated as a fold change (FC) between each gene against the geometric average of the chosen reference genes and calibrated to untreated controls. Genes reaching a relative gene expression level (fold change) < 0.5 or > 2 are considered as differentially expressed. The fold change ratio was calculated by dividing fold change value(s) of bacterial markers with that of viral markers.

### Statistical analysis

Statistical differences between experimental groups were determined using the Mann-Whitney test (Prism.5.03 Graph Pad Software).

## Results

### Selection of reference genes for expression analysis

The gene expression of *GAPDH*, *HPRT*, *PPIA*, *RPL32* and *YWHAZ*, in *in vitro* induced PBMC and in the clinical samples was determined and their stability evaluated using GeNorm software to find the best reference genes for normalization of expression. Overall, the *in vitro* inductions showed more stability between the reference genes (M = 0.486) than the clinical samples (M = 1.122) but the GeNorm M software ranked the reference genes rather similar in both types of samples (S1 Fig). The expression of each gene of interest was normalized against the three most stably expressed reference genes, i.e., *RPL32*, *PPIA* and *YWHAZ* for PBMC, and *RPL32*, *GAPDH* and *YWHAZ* for the clinical samples.

### Selection of bacterial and viral marker genes using TLR agonists

Porcine PBMC exposed to six different TLR agonists were screened for the expression of putative marker genes for bacterial or viral infections as well as genes indicative of TLR activation (Table 2). The upregulation of *IFN-β* by the viral mimics ODN 2216 and poly (I:C) and *IFN-α* by ODN 2216, as well as pro-inflammatory genes by the bacterial mimics (Pam3CSK4, LPS, FLiC), indicated that all inductions were successful. The putative viral markers *IFITM3*, *IFI44*, *MxA*, *RSAD2* were induced by viral but not bacterial mimics. In contrast, *IFIT3* was induced by the bacterial mimic LPS in addition to the viral mimics. Of the other interferon related genes, *IFITM3* was up-regulated by the viral mimics whereas the expression of *STING* remained unaffected. The putative bacterial markers *FAM89A* and *S100PBP* were unaffected or slightly downregulated by both viral and bacterial mimics. Furthermore, *UPB1* showed an upregulation by all inducers whereas *SLPI* was predominately upregulated by the viral mimics. On the other hand, the pro-inflammatory cytokines *IL-1β*, *IL-6* and *IL-8* were strongly upregulated by bacterial mimics and only to a lesser extent by viral mimics, varying with cytokine and inducer. Expression of TNF-α was low and with no preference between viral and bacterial inducers.

**Table 2:** Heat map illustrating gene expression patterns in porcine PBMC, exposed *in vitro* to ODN 2216, R848, poly (I:C), Pam3CSK4, FliC, LPS, split influenza virion or heat-inactivated *A*.*pleuropneumoniae* for 18h.

Category	Gene	Viral mimics			Bacterial mimics			Inactivated microbes	
		ODN 2216 (TLR9)	R848 (TLR7/8)	poly (I:C) (TLR3)	Pam3CSK4 (TLR2/1)	LPS (TLR4)	FLiC (TLR5)	Split Influenza	*A*. *pleuro* (HI)
Putative viral markers	*IFNα*	**3.4**	**0**	**1.0**	**0.2**	**0.3**	**0.3**	**1.7**	**0.4**
	*IFNβ*	**409.2**	**1.8**	**1992.0**	**3.0**	**0.5**	**1.7**	**70.9**	**1.4**
	*IFITM3*	**19.9**	**11.9**	**5.7**	**2.7**	**0.9**	**1.3**	**22.5**	**0.7**
	*STING*	**0.6**	**0.2**	**0.6**	**0.5**	**0.5**	**0.7**	**1.3**	**0.4**
	*IFI44L*	**7.5**	**3.4**	**3.3**	**1.4**	**0.7**	**1.1**	**8.0**	**0.5**
	*IFIT3*	**41.7**	**12.4**	**7.0**	**1.5**	**46.2**	**0.7**	**95.9**	**0.2**
	*MxA*	**72.6**	**39.5**	**17.7**	**7.9**	**0.5**	**1.0**	**63.0**	**0.7**
	*RSAD2*	**206.7**	**73.3**	**30.7**	**7.6**	**0.8**	**1.5**	**113.5**	**0,5**
Putative Bacterial markers	*FAM89A*	**0.6**	**0.1**	**0.5**	**0.5**	**0.8**	**0.8**	**0.6**	**0.6**
	*S100PBP*	**0.6**	**0.2**	**0.7**	**0.6**	**0.5**	**0.6**	**0.7**	**0.4**
	*SLPI*	**72.6**	**5.3**	**28.1**	**5.0**	**0.2**	**2.0**	**233.8**	**0.3**
	*UPB1*	**25.5**	**78.5**	**5.9**	**66.5**	**38.8**	**20.1**	**2.2**	**30.8**
Pro-inflam-matory cytokines	*IL-1β*	**1.0**	**1.8**	**10.3**	**67.2**	**40.7**	**30.0**	**0.7**	**17.7**
	*IL-6*	**20.7**	**96.7**	**19.7**	**72.3**	**51.7**	**24.5**	**3.7**	**12.2**
	*IL-8*	**1.9**	**9.0**	**44.0**	**119.8**	**66.6**	**32.0**	**0.3**	**24.3**
	*TNF-α*	**2.6**	**0.9**	**2.3**	**1.9**	**2.4**	**2.5**	**2.1**	**1.6**

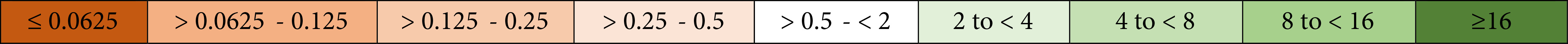

The gene expression was calculated as fold change in relation to the geometric average expression of three reference genes and calibrated to a medium control, using the average of duplicate reactions for each cDNA. Color denotes fold change expression range. Light to dark green denotes up-regulated and pink to orange denotes down-regulated genes.

### Selection of marker genes using inactivated bacterial and viral preparations

For further analysis, PBMC were exposed to heat-inactivated *A*. *pleuropneumoniae* or the split influenza virion and the expression of the putative viral, bacterial and pro-inflammatory marker genes was compared to the expression patterns induced by the TLR agonists. All viral markers except *STING* were clearly induced by the split influenza virion and down-regulated by the bacterial preparation (Table 2), in accordance with the viral TLR agonists. The viral markers *MxA*, *RSAD2*, *IFI44L* were taken for further analysis. *IFIT3* was excluded due to its upregulation by the bacterial mimic LPS.

Similar to what was observed for the TLR agonists, the putative bacterial markers *FAM89A* and *S100PBP* were unaffected by the viral and bacterial preparations (Table 2). *SLPI* was upregulated by the split influenza virion and down-regulated by the bacterial suspension whereas *UPB1* was up-regulated by both, albeit less by the split influenza virion. Consequently, *SLPI* and *UPB1* were discarded and only *FAM89A* and *S100PBP* were selected for further analyses as bacterial marker genes.

Of the pro-inflammatory cytokines, *IL-1β* and *IL-8* seemed to best discriminate between bacterial and viral inducers as they, albeit being induced by some viral mimics, were clearly up-regulated by FliC, LPS and heat-inactivated *A*. *pleuropneumoniae* but, down-regulated by the split influenza virion (Table 2). Based on this screening, four bacterial marker genes (*FAM89A*, *S100PBP*, *IL-1β*, *IL-8*) and three viral marker genes (*MxA*, *RSAD2*, *IFI44L)* were selected for further analyses.

### Selection of bacterial and viral marker genes based on their relative expression

**Fig 1:**
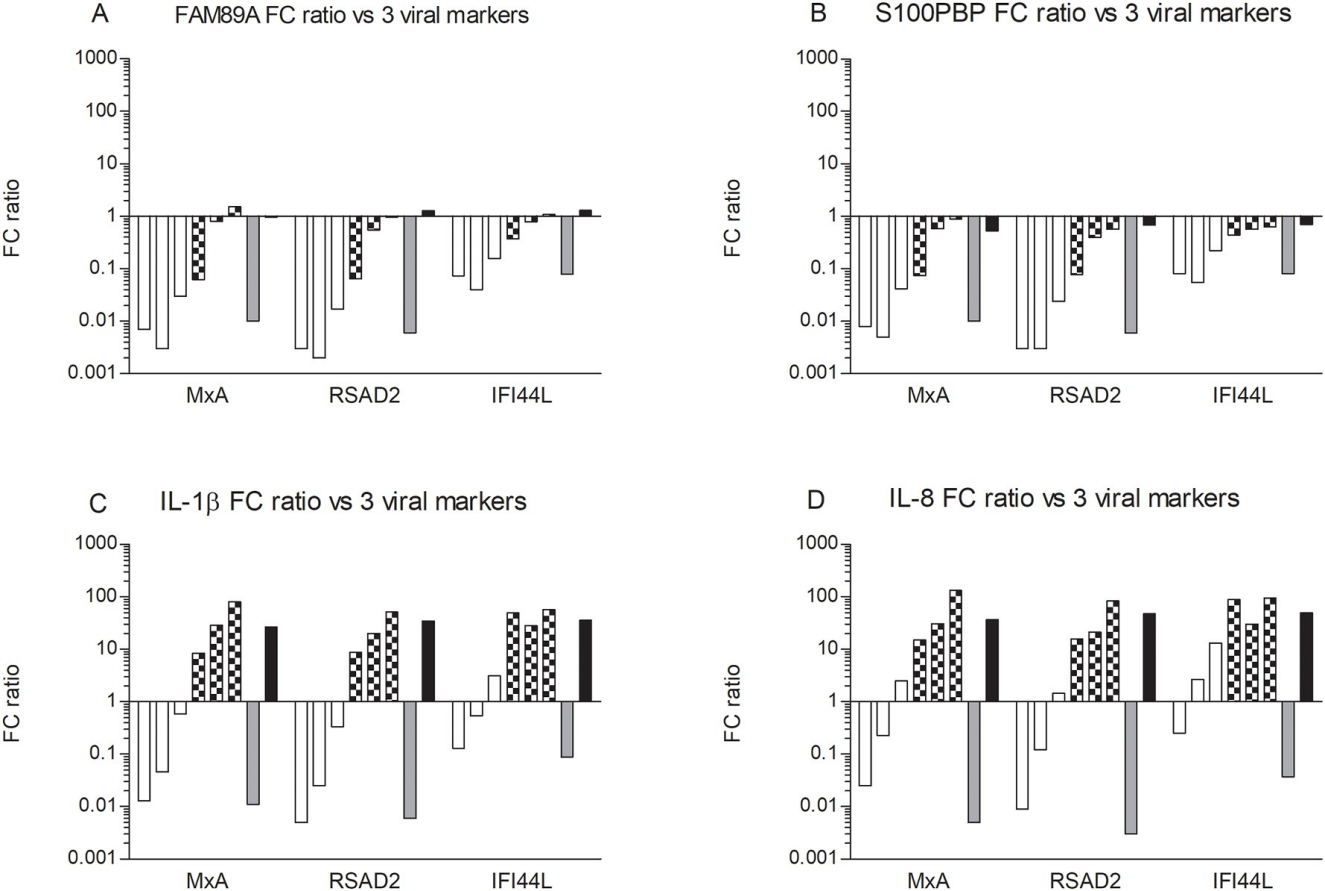
Fold change ratios of bacterial markers over viral markers in porcine PBMC. Fold change ratios were calculated as fold change of bacterial markers *FAM89A* (A), *S100PBP* (B), *IL-1β* (C) or *IL-8* (D) divided by fold change of the viral markers *MxA*, *RSAD2* and *IFI44L*, in porcine PBMC induced with ODN 2216, R848, poly (I:C), Pam3CSK4, FliC, LPS, split influenza virion or heat-inactivated *A*.*pleuropneumoniae* for 18h. Open bars from left to right (ODN 2216, R848, poly I:C), checkered bars (Pam3CSK4, FliC, LPS), grey bar (influenza virion), black bar (*A*. *pleuropneumoniae*).

To better assess the capacity of the putative bacterial marker genes to differentiate between bacterial and viral infections, a ratio between the fold change ratio for each of the bacterial markers (*FAM89A*, *S100PBP*, *IL-1β*, *IL-8*) to each of three viral markers (*MxA*, *RSAD2*, *IFI44L*) was calculated (Fig 1). All four bacterial markers showed a reduced fold change ratio when the PBMC were exposed to the influenza virion. The fold change ratios for both *FAM89A* and *S100PBP* remained unaffected when the PBMC were exposed to *A*. *pleuropneumoniae* (Fig 1A and 1B), whereas IL-1β and IL-8 showed an increased fold change ratio (Fig 1C and 1D). This pattern was also evident in the inductions with different TLR agonists, with the exception of Pam3CSK4 which down-regulated both FAM89A and S100PBP similar to the viral inducers. The fold change ratios showed a similar pattern regardless of the viral marker used.

### Evaluation of candidate markers in clinical samples

**Fig 2:**
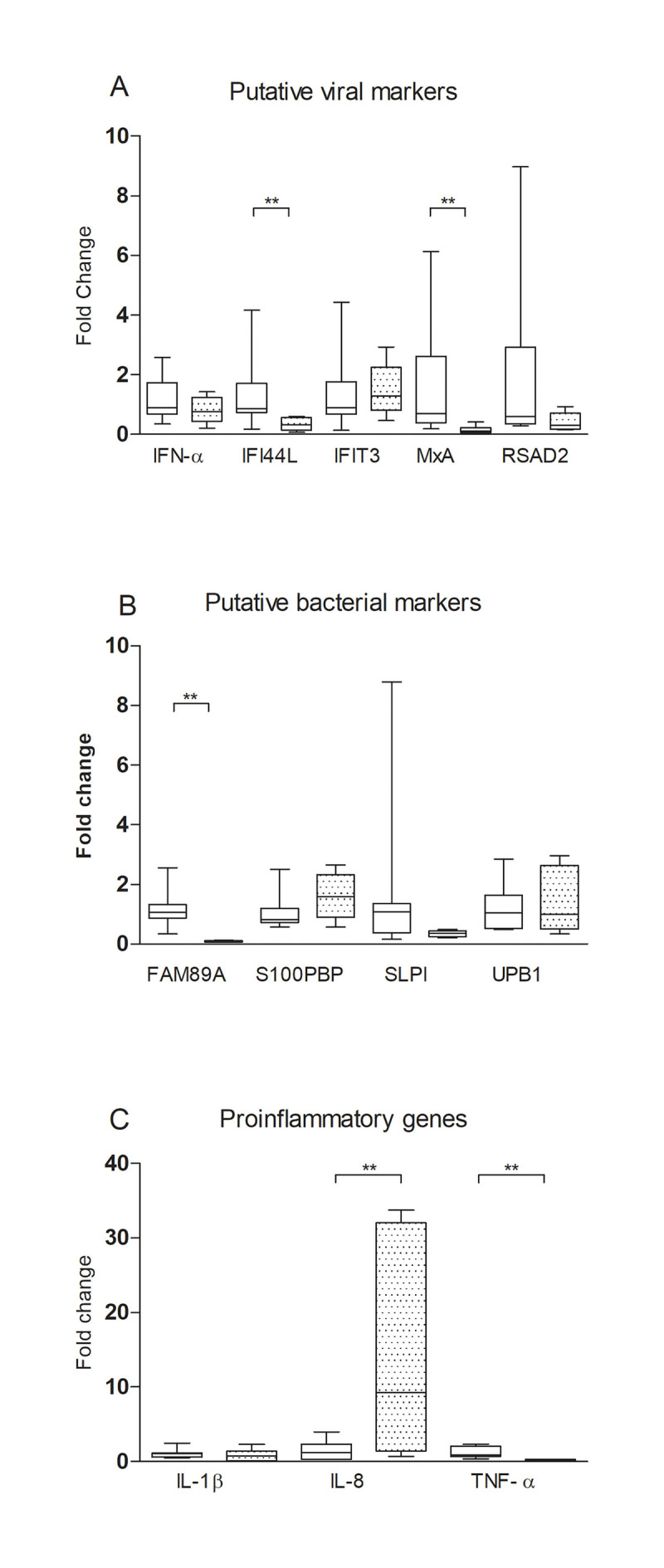
Expression of potential markers in *A*. *pleuropneumoniae* infected pigs. The Fold change denotes the expression of potential marker genes in SPF pigs (n = 11) and pigs infected with *A*. *pleuropneumoniae* (n = 6) normalized to the geometric average expression of the three reference genes *RPL32*, *GAPDH* and *YWHAZ*. The expression in each pig was calibrated to that of the average expression in the SPF pigs and is shown as box plots. * p<0.05, ** p<0.01.

The expression of the putative viral (*IFI44L*, *IFIT3*, *MxA*, *RSAD2*), bacterial (*FAM89A*, *UPB1*, *S100PBP*, *SLPI*) and pro-inflammatory (*TNF-α*, *IL-1β*, *IL-6*, *IL-8)* marker genes was also determined in samples encompassing eleven SPF pigs and six pigs infected two weeks earlier with *A*. *pleuropneumoniae* (Fig 2).

The expression of the putative viral markers IFI44L and MxA was significantly down-regulated in infected pigs compared to SPF pigs (Fig 2A). Of the putative bacterial markers only S100PBP indicated a tendency to up-regulation in infected pigs but it was not statistically significant (Fig 2B). IL-8 was the only proinflammatory gene that showed a significant up-regulation in infected pigs (Fig 2C). The most prominent finding in the *A*. *pleuropneumoniae* infected pigs was a consistent down-regulation of the putative viral marker genes *IFI44L* and *MxA* and an up-regulation of *IL-8*.

**Fig 3:**
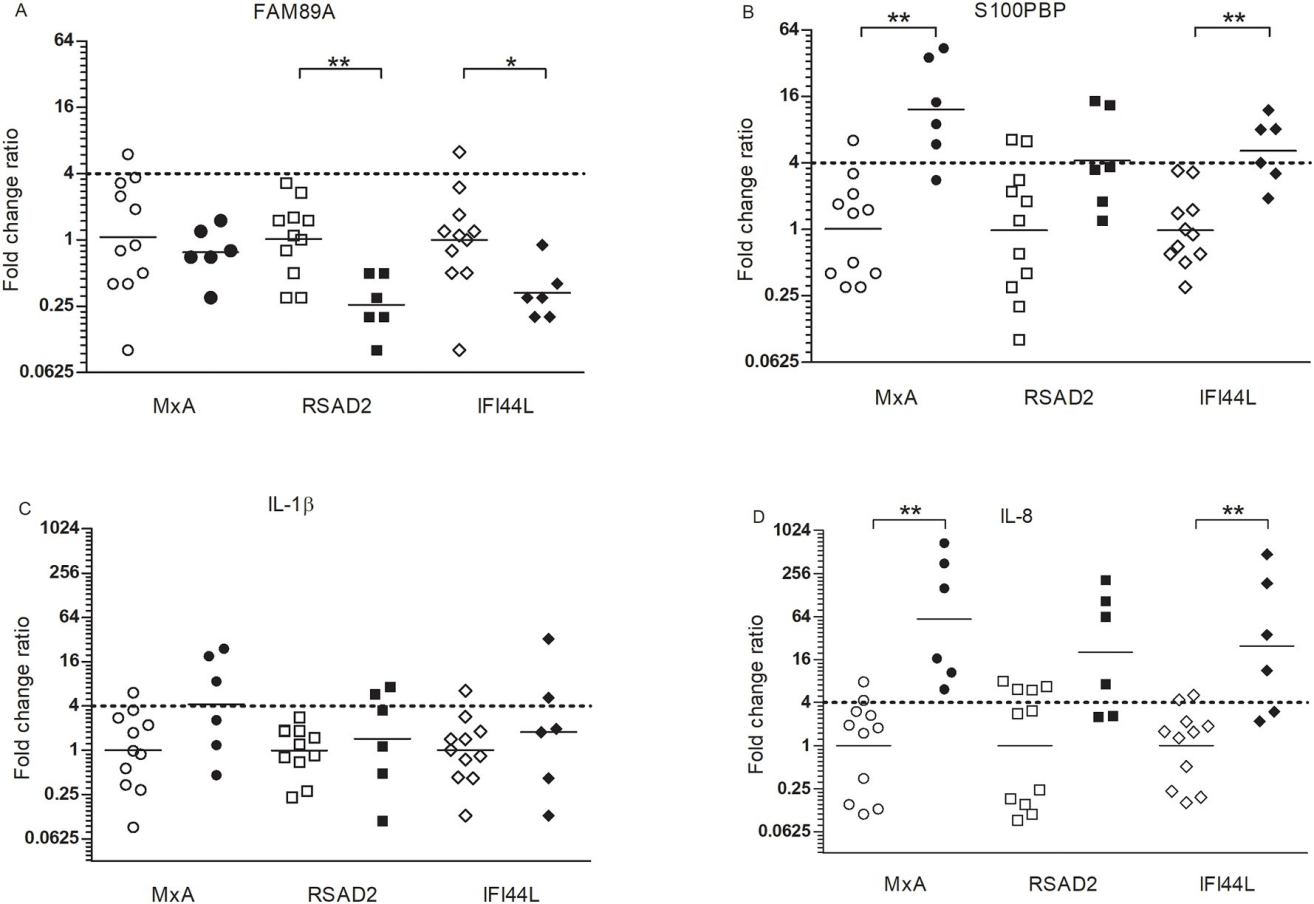
Fold change ratios in *A*. *pleuropneumoniae* infected pigs. Fold change ratios were calculated as fold change of bacterial markers *FAM89A* (A), *S100PBP* (B), *IL-1β* (C) or *IL-8* (D) divided by fold change of the viral markers *MxA*, *RSAD2* and *IFI44L* in eleven SPF pigs (open symbols) and six pigs with an *A*. *pleuropneumoniae* infection (filled symbols). Fold change ratio = 4 is depicted by the dotted line. * p<0.05, ** p<0.01.

Further evaluation of *FAM89A*, *S100PBP*, *IL-1β* or *IL-8* in relation to *MxA*, *RSAD2* or *IFI44L* as fold change ratios revealed that the bacterial marker candidate FAM89A varied the most in expression in the SPF pigs and was even reduced in the infected pigs (Fig 3A). The fold change ratios of IL-1β showed no statistically significant difference between healthy and infected pigs regardless of viral marker used (Fig 3C). The expression ratios of *S100PBP* or *IL-8* showed a statistically significant difference between SPF and diseased pig when using either MxA or IFI44L as viral marker (Fig 3B and 3D). Furthermore, when using MxA as a viral marker, the best separation of SPF and diseased pigs was achieved. Setting a cut off of 4 for the fold change ratio of *S100PBP* allowed the best differential resolution, scoring bacterial infection in 5 of the 6 infected pigs, whereas only one healthy pig was scored as a false positive for bacterial infection (Fig 3B). The same was true for IL-8, scoring six out of six pigs as infected, but also two of the healthy pigs (Fig 3D).

**Fig 4:**
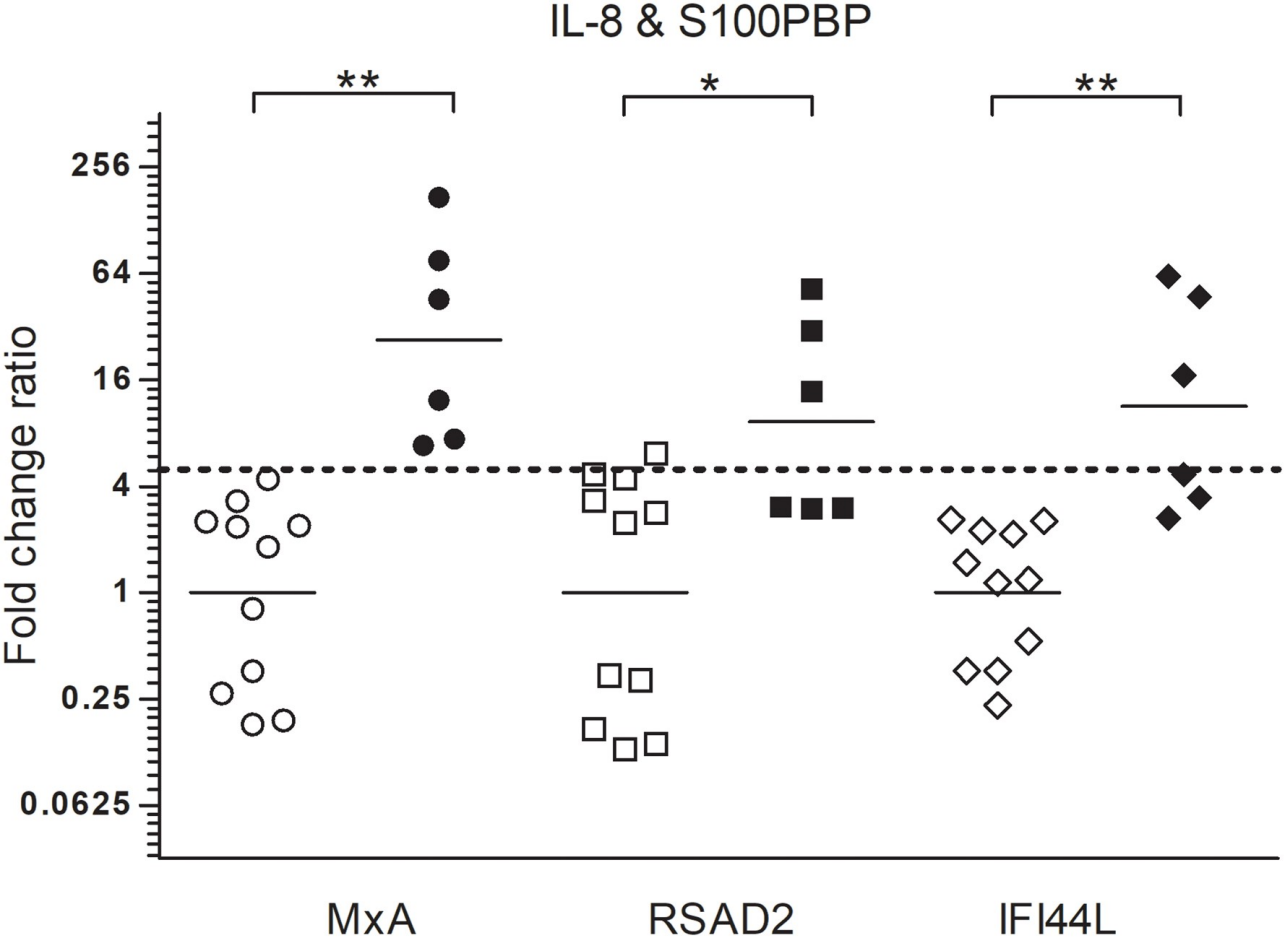
Combined fold change ratios using both *S100PBP* and *IL-8*. The geometric average of fold change ratios of the bacterial markers *S100PBP* and *IL-8* versus viral markers *MxA*, *RSAD2* or *IFI44L* was calculated for eleven SPF pigs (open symbols) and six pigs with an *A*. *pleuropneumoniae* infection (filled symbols). Fold change ratio = 5 is depicted by the dotted line. * p<0.05, ** p<0.01.

Since not the same SPF pigs scored false positive using the two bacterial markers, *S100PBP* and *IL-8* were combined in an attempt to improve the diagnostic power. Therefore, the geometric average fold change of both genes versus any of the three viral markers were analysed (Fig 4). The combination of these two markers showed a statistically significant difference between SPF pigs and infected pigs with any of the viral markers used. However, using the fold change ratio of both bacterial markers versus *MxA* and a cut-off fold change ratio of 5, separated all infected animals from the SPF pigs.

## Discussion

The combination of three genes, MxA (viral), IL-8 (bacterial) and S100PBP (bacterial) in a quota, turned out as a potential read-out for differentiation between viral and bacterial infections in pigs. The results narrowed down to this combination of transcripts after evaluating a number of genes previously proposed to discriminate between viral and bacterial infections in human patients.

The immune response to viral or bacterial exposure broadly triggers interferon (IFN)-related and integrin-related signatures, respectively [26]. Frequently evaluated human biomarkers include C reactive protein (CRP), Procalcitonin (PCT), IL-6 and IL-8 for bacterial infections and tumor necrosis factor-related apoptosis-inducing ligand (TRAIL), interferon (IFN)-γ -induced protein-10 (IP-10) and myxovirus resistance protein A (MxA) for viral infections. We chose to evaluate the porcine counterparts to some of the most promising markers identified in the study by Herberg et al. [19], and the pro-inflammatory genes IL-1β, IL-6, IL-8 and TNF-α. Furthermore, MxA was included among the markers to be evaluated as it has already been applied in a commercial test for diagnostics in humans [27] and is readily expressed in the pig [28]. On the other hand, S100P showed promise as a bacterial marker in humans but seems not to be present in pigs. However, its interacting partner in humans, S100PBP, is present in pigs and was therefore chosen for evaluation as a bacterial marker.

In the first selection step, porcine PBMC were induced with a number of TLR agonists and the expression of each marker was analyzed. The induced gene expression via a single PRR probably does not reflect very well the induction pattern of a complete pathogen being recognized by many PRRs. Furthermore, there is considerable variation in TLR responsiveness to specific agonists between different species and even breeds [29]. This might explain why R848, a strong inducer of type I IFNs in humans and mice, failed to induce them in our induction. However, the combined results from inductions with a range of agonists might mimic an *in vivo* infection better. This seems true for many of the markers, especially the viral ones (IFI44L, MxA, RSAD2) when evaluated using PBMC exposed to either heat-killed *Actinobacillus pleuropneumoniae* or split influenza virus.

The *in vitro* studies were conducted with PBMC isolated from SPF-pigs and their response to the TLR agonists was always related to the expression of genes in parallel cultures of the PBMC in plain medium. For *in vivo* studies, the gene expression of eleven SPF-pigs was used as a reference and combined to that of six pigs in the late phase (day 16) of an experimental infection with *A*. *pleuropneumoniae*. During the acute phase of the infection these pigs all displayed clinical symptoms, elevated serum amyloid A levels, a febrile response and neutrophilia. Somewhat surprisingly four of the six pigs showed high expression of the gene for IL-8 and all six pigs showed a markedly depressed expression of *MxA*. This reversed relationship is in line with the finding that IL-8 can interfere with 2′, 5′-A oligoadenylate synthetase activity, also induced via an IFN-α/β regulated pathway [30]. Thus, the combined use of IL-8 and MxA is likely to magnify a bacterial gene expression signature in the pig. Human MxA and IL-8 have received much attention as possible markers for viral and bacterial infections, respectively [12]. MxA has even been implemented in a commercial lateral flow assay, FebriDx, simultaneously detecting MxA and CRP for use in patients with acute respiratory infections [27]. The S100 proteins are a family of calcium-binding cytosolic proteins important for regulating inflammation [31]. The S100P-binding protein, S100PBP, has been shown to downregulate Cathepsin Z in an S100P-independent manner [32]. Cathepsin Z, in turn, can suppress lymphocyte proliferation via activation of the β_2_ integrin Mac-1 on macrophages [33]. That is, upregulation of S100PBP could, in the end, lead to increased lymphocyte proliferation. Several S100 proteins seem to belong to the same expression signature as IL-8, i.e. IL-8 can induce the expression of S100A8/A9 and S100A12 can induce the expression of IL-8 [31]. Interestingly, S100A12 was identified as an inflammatory biomarker when porcine whole blood was exposed to LPS [34]. For future work, these genes might be suitable as markers for bacterial infection together with IL-8.

The two genes comprising the human two-transcript signature IFI44L/FAM89A [19] did not work as well on our material. FAM89A showed no promise as a bacterial marker in either *in vitro* induced PBMC or clinical material and MxA showed better differential results than IFI44L. In a further evaluation of the human two-transcript, it was concluded that IFI44L alone yielded better results than when combined with FAM89A [35]. To some extent, this effect can also be seen with MxA on our material, although the full differential result was only achievable in combination with IL-8 and S100PBP. The combination of several markers might be valuable to accomplish a better diagnostic power and extend the window in which an effect of an infection could be seen in the transcriptional response of the host. For instance, the first host response gene expression assay for diagnosis of sepsis, SeptiCyte LAB, is based on the four marker genes CEACAM4, LAMP1, PLAC8 and PLA2G7 [36]. In the present study, S100PBP showed no differential expression in the *in vitro* inductions but was nevertheless slightly upregulated in the infected pigs. In view of this, *in vitro* inductions could be a useful tool to evaluate the kinetics of expression of putative marker genes. Furthermore, any assay based on a single transcript would necessitate the use of reference genes adding the complexity of having to evaluate and select these too. Using a score of several marker genes, both bacterial and viral, for a discriminating diagnosis can circumvent this, albeit that a carefully chosen control or cut-off point is still necessary.

Results presented in this pilot study constitute a first attempt to design a host gene differential expression assay for the pig and should in the future be evaluated further on more clinical material comprising single and co-infections, parasitic infections and vaccinated animals. Further marker choices should also be explored and the kinetics of expression determined.

In conclusion, here we present a minimal transcript signature with the potential to distinguish bacterial from viral diseases and thereby increase the precision in therapeutic means, ultimately reducing the use of antibiotics in pig rearing.

## Supporting information

S1 Fig GeNorm ranking of reference genes. Expression stability of five reference genes in porcine PBMC induced with ODN 2216, R848, poly (I:C), Pam3CSK4 or FliC (top) or eleven healthy SPF pigs and six pigs infected with *A*. *pleuropneumoniae* (bottom) was evaluated using qBasePLUS software (Biogazelle).
(DOCX)

S1 Table Sequences and assay conditions of primer pairs. (DOCX)
