# Supplementary material for: Development of a 3-transcript host expression assay to differentiate between viral and bacterial infections in pigs
Source: PLoS One. 2021 Sep 23;16(9):e0256106. doi: 10.1371/journal.pone.0256106 (PMC8459988; doi:10.1371/journal.pone.0256106)
Supplement: S1 Table — (DOCX) [file pone.0256106.s002.docx]

| **Gene** | **Sequence** | **Concent mM** | **Anneal Temp (C°)** | **Eff (%)^a^** | **r2** | **Melt point (C°)** | **Product length (bp)** | **References** |
| --- | --- | --- | --- | --- | --- | --- | --- | --- |
| *GAPDH* | Fw 5´ ACACTCACTCTTCTACCTTTG 3´ | 500 | 56 | 94 | 0,998 | 79 | 90 | [1] |
|  | Rv 5´ CAAATTCATTGTCGTACCAG 3´ |  |  |  |  |  |  |  |
| *HPRT* | Fw 5´ GGTCAAGCAGCATAATCCAAAG 3´ | 500 | 60 | 100 | 0,996 | 79,5 | 134 | [2] |
|  | Rv 5´ CAAGGGCATAGCCTACCACAA 3´ |  |  |  |  |  |  |  |
| *PPIA* | Fw 5´ GCAGACAAAGTTCCAAAGACAG 3´ | 400 | 60 | 92 | 0,999 | 79,5 | 217 | [3] |
|  | Rv 5´ AGATGCCAGGACCCGTATG 3´ |  |  |  |  |  |  |  |
| *RPL32* | Fw 5´ TGGAAGAGACGTTGTGAGCAA 3´ | 500 | 56 | 97 | 0,997 | 76,5 | 93 | [4] |
|  | Rv 5´ CGGAAGTTTCTGGTACACAATGTAA 3´ |  |  |  |  |  |  |  |
| *YWHAZ* | Fw 5´ ATTGGGTCTGGCCCTTAACT 3´ | 400 | 58 | 101 | 0,997 | 78 | 146 | [4] |
|  | Rv 5´ GCGTGCTGTCTTTGTATGACTC 3´ |  |  |  |  |  |  |  |
| *CRP* | Fw 5´ AGAAGCTGTCACTGTGTCTCC 3´ | 400 | 55 | Nd | Nd | Nd | 75 | New design |
|  | Rv 5´ GCCTTTCCGATCATGTCTGTC T 3´ |  |  |  |  |  |  |  |
| *FAM89A* | Fw 5´ TGCGTAAAGAGATGGTCGGC 3´ | 500 | 60 | 97 | 0,994 | 81,5 | 97 | New design |
|  | Rv 5´ CACGCTCCCTTGTACTCCTG 3´ |  |  |  |  |  |  |  |
|  | Fw 5´CTACGCGCAGAAGTCTCGG 3´ | 400 | 55 | D | D | D | 142 | New design |
|  | Rv 5´ GAGGCCGACCATCTCTTTACG 3 |  |  |  |  |  |  |  |
| *IFI44L* | Fw 5´ ATCCAACTCTGAAAACAGATACGG 3´ | 400 | 58 | 96 | 0,981 | 76,5 | 177 | New design |
|  | Rv 5´ AGTGGATCCCTGACAACTGC 3´ |  |  |  |  |  |  |  |
|  | Fw 5´ GCCACACCGCACAGAGATAG 3´ | 400 | 55 | D | D | D | 104 | New design |
|  | Rv 5´ GACCCAACTGGACCCAACAA 3´ |  |  |  |  |  |  |  |
| *IFI27* | Fw 5´GGGTCCCCATGATGAAACGG 3´ | 400 | 55 | Dd | Dd | Dd | 140 | New design |
|  | Rv 5´ TGACATCATCTTGGCCGCTA 3´ |  |  |  |  |  |  |  |
|  | Fw 5´ GTCCCCATTGGTTGTCGGG 3´ | 400 | 55 | Dd | Dd | Dd | 102 | New design |
|  | Rv 5´ GCTGCCCGTTTCATCATGG 3´ |  |  |  |  |  |  |  |
| *IFIT1* | Fw 5´ GCTGCCCGTTTCATCATGG 3´ | 400 | 55 | Dd | Dd | Dd | 141 | New design |
|  | Rv 5´ CTCAATCTCCTCCAAGACCCTG 3´ |  |  |  |  |  |  |  |
| *IFIT3* | Fw 5´ CCGCCATCATGAGTGAGGTC 3´ | 500 | 60 | 93 | 0,991 | 78 | 136 | New design |
|  | Rv 5´ TCAGTCTGGTTACACACTCTGTC 3´ |  |  |  |  |  |  |  |
| *IFITm3* | Fw 5´ ATCAACATCCGAAGCGAGACC 3´ | 500 | 56 | 96 | 0,999 | 85,5 | 276 | [5] |
|  | Rv 5´ GGAAAATTACCAGGGAGCCAGTG 3´ |  |  |  |  |  |  |  |
| *IFNA* | Fw 5´ AGCCTCCTGCACCAGTTCTG 3´ | 500 | 60 | 100 | 0,997 | 84,5 | 124 | [4] |
|  | Rv 5´ TCACAGCCAGGATGGAGTCC 3´ |  |  |  |  |  |  |  |
| *IFNB* | Fw 5´ TCAGGTGAAGAATGGTCATGTCT 3´ | 400 | 58 | 104 | 0,993 | 79,5 | 140 | [4] |
|  | Rv 5´ TAGCACTGGCTGGAATGAAACC 3´ |  |  |  |  |  |  |  |
| *IL-1B* | Fw 5´ GTGATGGCTAACTACGGTGACAA 3´ | 400 | 60 | 91 | 0,999 | 79,5 | 113 | [6] |
|  | Rv 5´ CTCCCATTTCTCAGAGAACCAAG 3´ |  |  |  |  |  |  |  |
| *IL-6* | Fw 5´ CTGGCAGAAAACAACCTGAACC 3´ | 400 | 60 | 98 | 0,994 | 77,5 | 94 | [7] |
|  | Rv 5´ TGATTCTCATCAAGCAGGTCTCC 3´ |  |  |  |  |  |  |  |
| *IL-8* | Fw 5´ AGCCAGGAAGAGACTAGAAAGAAA 3´ | 500 | 56 | 97 | 0,998 | 81,5 | 179 | [5] |
|  | Rv 5´ TTGGGGTGGAAAGGTGTG 3´ |  |  |  |  |  |  |  |
| *MxA* | Fw 5´ CAGAGGCAGCGGAATTGTG 3´ | 500 | 58 | 103 | 0,976 | 79 | 109 | New design |
|  | Rv 5´ AATCTCGCTGTCCCGGTAAC 3´ |  |  |  |  |  |  |  |
|  | Fw 5´ CAGAGGCAGCGGAATTGTGA 3´ | 400 | 55 | D | D | D | 99 | New design |
|  | Rv 5´ TCCCGGTAACTGACTTTGCC 3´ |  |  |  |  |  |  |  |
| *OTOF* | Fw 5´ GCTATTCGACTGGGGGATGG 3´ | 400 | 55 | Nd | Nd | Nd | 97 | New design |
|  | Rv 5´ CTGGTTTGGACCGCTTGT TG 3´ |  |  |  |  |  |  |  |
|  | Fw 5´ ACATCGAGTGTGCAGGGAAG 3´ | 400 | 55 | Nd | Nd | Nd | 104 | New design |
|  | Rv 5´ GGGGAGGTCCACTTCAAACC 3 |  |  |  |  |  |  |  |
| *PI3* | Fw 5´ CTGGCAGAATACGTCGTC CT 3´ | 400 | 55 | Dd | Dd | Dd | 81 | New design |
|  | Rv 5´ TACCTCTCACCGGACCTTGT 3´ |  |  |  |  |  |  |  |
| *PTPN20* | Fw 5´ TCAAGTTGTCAGGAAGTCTACAGG 3´ | 400 | 55 | Nd | Nd | Nd | 149 | New design |
|  | Rv 5´ TCAAGTTGTCAGGAAGTCTACAGG 3´ |  |  |  |  |  |  |  |
|  | Fw 5´ GCTGTGTTCCACTCTGGGAA 3´ | 400 | 55 | Nd | Nd | Nd | 83 | New design |
|  | Rv 5´ AACACGTGTTGAATCATATGGAAG 3´ |  |  |  |  |  |  |  |
| *RSAD2* | Fw 5´ GGACGTGGTGCAGGGATTAT 3´ | 300 | 60 | 95 | 0,998 | 78 | 123 | New design |
|  | Rv 5´ GGAAGACCTTCCAGCGGACA 3´ |  |  |  |  |  |  |  |
|  | Fw 5´ TGAAGGAAGCGGGTATGGAG 3´ | 400 | 55 | D | D | D | 102 | New design |
|  | Rv 5´ CCTCCTTGCAGAACCTGACC 3´ |  |  |  |  |  |  |  |
| *S100PBP* | Fw 5´ TTCCAGTTCATCAAACAAAGATGTTC 3´ | 500 | 58 | 99 | 0,883 | 78 | 136 | New design |
|  | Rv 5´ ATCTGATGGTGAAGACGTCGG 3´ |  |  |  |  |  |  |  |
| *SLPI* | Fw 5´ CAAGTGCACAAGTGACTGGC 3´ | 500 | 60 | 93 | 0,998 | 79,5 | 113 | New design |
|  | Rv 5´ CAGGCTTCACCTTAACTGGGT 3´ |  |  |  |  |  |  |  |
|  | Fw 5´ GAACCCAGTTAAGGTGAAGCC 3´ | 400 | 55 | D | D | D | 94 | New design |
|  | Rv 5´ CTGGCTGTCTGTCTTGCAGT 3´ |  |  |  |  |  |  |  |
| *STING* | Fw 5´ TTACATCGGGTACCTGCGGC 3´ | 500 | 56 | 101 | 0,992 | 81 | 84 | [4] |
|  | Rv 5´ CCGAGTACGTTCTTGTGGCG 3´ |  |  |  |  |  |  |  |
| *TMEM119* | Fw 5´ CAGACGTGCCATCTGAGGAG 3´ | 400 | 55 | Nd | Nd | Nd | 80 | New design |
|  | Rv 5´ TAACAAGGGAGCCGCTTCTG 3´ |  |  |  |  |  |  |  |
|  | Fw 5´ AAGGAACTGGTCCTGGGCAAC 3´ | 400 | 55 | Nd | Nd | Nd | 108 | New design |
|  | Rv 5´CAGGGACACGGAGTAGGACG 3´ |  |  |  |  |  |  |  |
| *TNFA* | Fw 5´ AGCCTCTTCTCCTTCCTCCTG 3´ | 400 | 60 | 91 | 0,993 | 83 | 145 | [8] |
|  | Rv 5´ GAGACGATGATCTGAGTCCTTGG 3´ |  |  |  |  |  |  |  |
| *UPB1* | Fw 5´ TGCCAATCACTGCTTCACCT 3´ | 500 | 60 | 88 | 0,994 | 81 | 102 | New design |
|  | Rv 5´ CCGAAGTCCTGGTGAGCTTT 3´ |  |  |  |  |  |  |  |
|  | Fw 5´ CCTGTGGTGAAACAGGTCACT 3´ | 400 | 55 | D | D | D | 101 | New design |
|  | Rv 5´ CACGCCTCCTGGAAACAGAT 3´ |  |  |  |  |  |  |  |

^a^ based on serial dilutions of reference cDNA; Nd Not detected and therefore discarded

D Discarded in favor of the other assay; Dd Detected but discarded in favor of other markers

1. Nygard AB, Jorgensen CB, Cirera S, Fredholm M. Selection of reference genes for gene expression studies in pig tissues using SYBR green qPCR. BMC Mol Biol 2007; 8:67.
2. Feng X, Xiong Y, Qian H, Lei M, Xu D, Ren Z. (2010). Selection of reference genes for gene expression studies in porcine skeletal muscle using SYBR green qPCR. J Biotechnol. 2010; 150: 288-293.
3. McCulloch RS, Ashwell MS, O’Nan AT, Mente PL. Identification of stable normalization genes for quantitative real-time PCR in porcine articular cartilage. J Anim Sci Biotechnol. 2012; 3:36.
4. Fossum C, Hjertner B, Ahlberg V, Charerntantanakul W, McIntosh K, Fuxler L, et al. Early inflammatory response to the saponin adjuvant Matrix-M in the pig. Vet Immunol Immunopathol 2014; 158:53-61.
5. Ahlberg V, Hjertner B, Wallgren P, Hellman S, Lövgren Bengtsson K, Fossum, C. Innate immune responses induced by the saponin adjuvant Matrix-M in specific pathogen free pigs. Vet Res 2017;48: 30.
6. Shirkey TW, Siggers RH, Goldade BG, Marshall JK, Drew MD, Laarveld B. Effect of commensal bacteria on intestinal morphology and expression of proinflammatory cytokines in the gnotobiotic pig. Exp Biol med 2006; 231:1333-1345.
7. Duvigneau JC, Hartl RT, Groiss S, Gemeiner M. Quantitative simultaneous multiplex real-time PCR for the detection of porcine cytokines. J immunol methods 2005; 306:16-27.
8. Wikström FH, Fossum C, Fuxler L, Kruse R, Lövgren T. Cytokine induction by immunostimulatory DNA in porcine PBMC is impaired by a hairpin forming sequence motif from the genome of Porcine Circovirus type 2 (PCV2). Vet Immunol Immunopathol 2011; 139:156-166.
