# Supplementary material for: Development of a 3-transcript host expression assay to differentiate between viral and bacterial infections in pigs
Source: PLoS One. 2021 Sep 23;16(9):e0256106. doi: 10.1371/journal.pone.0256106 (PMC8459988; doi:10.1371/journal.pone.0256106)

Ranking of reference genes in induced porcine PBMC

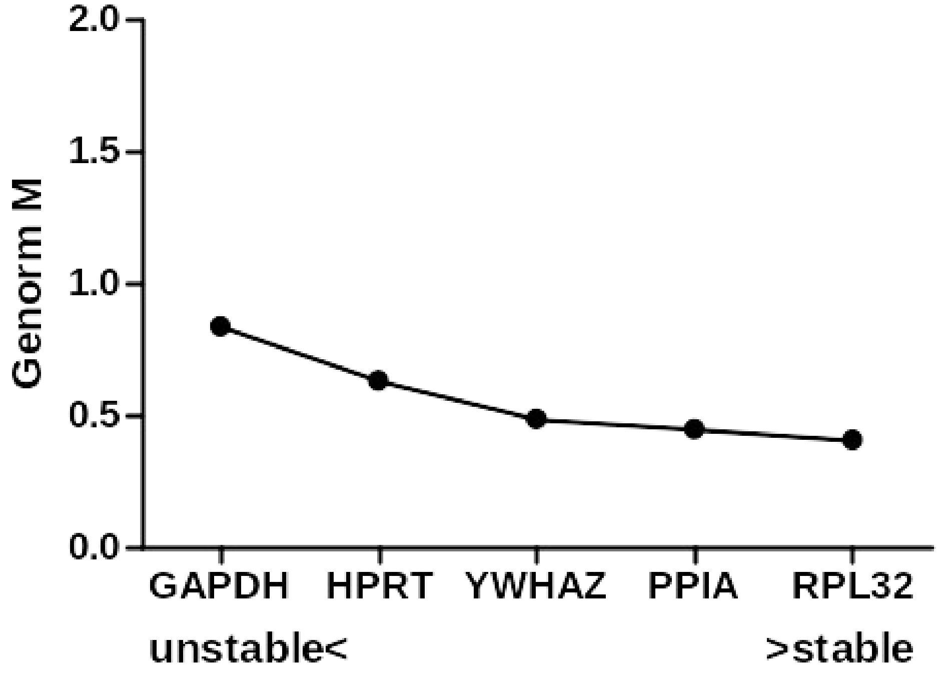

Ranking of reference genes in healthy and infected pigs

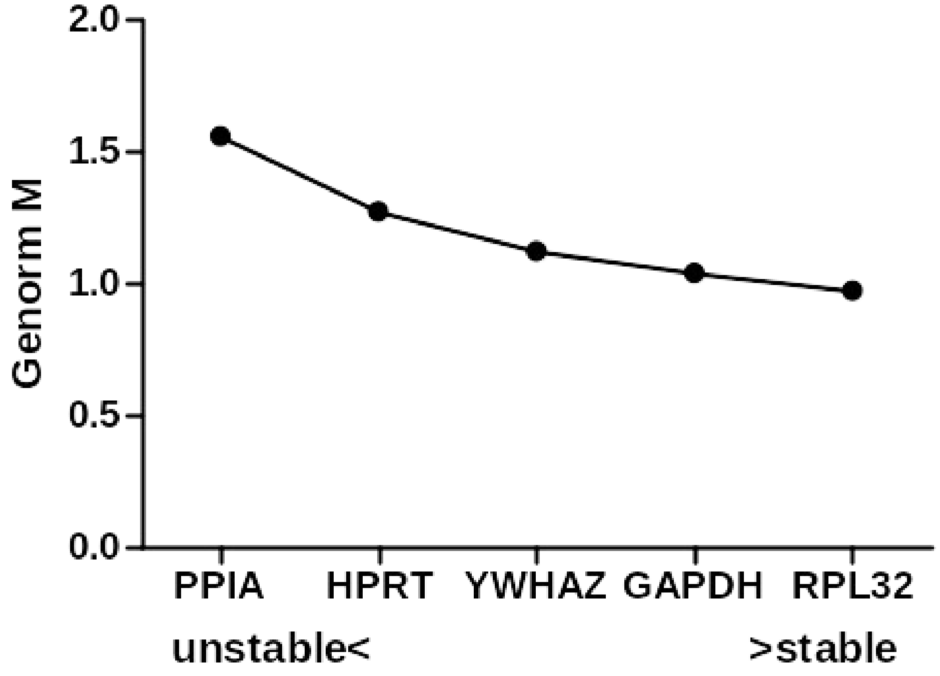

Supplement: Supplementary file 2 [file pone.0256106.s001.docx]
